# Developing a suicide risk prediction model for hospitalized adolescents with depression in China

**DOI:** 10.3389/fpsyt.2025.1532828

**Published:** 2025-05-02

**Authors:** Juan Zhao, Ying Li, Yangjie Chen, Ahmad Naqib Shuid

**Affiliations:** ^1^ Department of Psychiatry, First Hospital / First Clinical Medical College of Shanxi Medical University, Taiyuan, China; ^2^ 2Advanced Medical and Dental Institute, Universiti Sains Malaysia, Kepala Batas, Pulau Pinang, Malaysia; ^3^ Senior Department of Orthopedics, The Fourth Medical Center of People's Liberation Army (PLA) General Hospital, Beijing, China

**Keywords:** adolescent depression, suicide risk, self-harm, parental relationship, social support, sleep duration, prediction model

## Abstract

**Introduction:**

Adolescent suicide risk, particularly among individuals with depression, is a growing public health concern in China, driven by increasing social pressures and evolving family dynamics. However, limited research has focused on suicide prediction models tailored for hospitalized Chinese adolescents with depression. This study aims to develop a suicide risk prediction model for early identification of high-risk individuals using internal validation, providing insights for future clinical applications.

**Methods:**

The study involved 229 adolescents aged 13–18 diagnosed with depression, admitted to a hospital in Shanxi, China. Feature selection was performed using Least Absolute Shrinkage and Selection Operator (Lasso) regression, and key predictors were incorporated into a multivariate logistic regression model. Model performance was assessed using the area under the receiver operating characteristic curve (AUC), Hosmer-Lemeshow test, calibration curves, decision curve analysis (DCA), and clinical impact curves (CIC).

**Results:**

The model demonstrated AUC values of 0.839 (95% CI: 0.777, 0.899) for the training set and 0.723 (95% CI: 0.601, 0.845) for the testing set, indicating strong discrimination capability. Significant predictors included gender, social frequency, parental relationships, self-harm behavior, experiences of loss, and sleep duration. DCA and CIC supported the model’s predictive potential.

**Conclusion:**

The model demonstrated strong predictive performance in internal validation, suggesting potential value for suicide risk assessment in hospitalized adolescents with depression. However, its generalizability remains to be confirmed. Further external validation in larger, multi-center cohorts is required to assess its robustness and clinical applicability.

## 1 Introduction

Adolescence represents a critical developmental stage between childhood and adulthood, characterized by profound biological, psychological, and social transformations that increase susceptibility to mental health disorders (1, 2). This period is marked by heightened emotional reactivity and neurobiological changes, which contribute to an increased risk of psychiatric conditions, particularly depression (3, 4). In recent years, the prevalence of adolescent depression has risen significantly worldwide, with China experiencing a particularly concerning increase. This surge is largely attributed to rapid social development and evolving family structures, which have intensified mental health challenges among young individuals (5). Epidemiological studies estimate that the lifetime prevalence of depression among Chinese adolescents has reached 24.3%, aligning with global trends (6). Beyond its profound emotional toll, depression is a major risk factor for suicide, with many affected adolescents experiencing suicidal ideation or attempting suicide (7). A meta-analysis reported that 38.2% of adolescents diagnosed with depression engage in suicidal ideation or suicide attempts (8). According to the World Health Organization (9), suicide has become the fourth leading cause of death among individuals aged 15 to 24 worldwide, underscoring the urgent need for effective risk assessment and prevention strategies (9).

Adolescents with depression face a disproportionately high risk of suicide, particularly in psychiatric inpatient settings. Studies indicate that the suicide risk for psychiatric inpatients is up to 50 times higher than that of the general population, with depression being a key vulnerability factor (10). Many of these patients have a history of suicide attempts and self-harm, both of which are robust predictors of future suicide risk (11). Self-harming behavior is especially prevalent among hospitalized adolescents with depression and is regarded as a critical warning sign for potential suicidal behavior (12). Furthermore, research highlights that the first week of hospitalization is a particularly high-risk period, underscoring the urgency of early identification and intervention (13). Accurate identification of high-risk adolescents in psychiatric inpatient settings, along with effective risk management strategies, remains a significant challenge for mental health professionals. Addressing this issue necessitates data-driven predictive models that can enhance suicide risk stratification and guide timely intervention efforts.

Traditional approaches to suicide risk assessment in adolescents with depression exhibit several limitations. Firstly, these methods frequently rely on subjective clinical judgment, which can be highly variable and inconsistent, resulting in discrepancies in identifying high-risk individuals (14). Secondly, suicide risk is multifaceted, involving complex interactions among social, familial, and psychological factors (15). Traditional screening tools struggle to effectively process and integrate these multidimensional data, thereby limiting their predictive accuracy (16). Furthermore, healthcare professionals frequently operate under significant time and resource constraints, making it challenging to conduct comprehensive suicide risk assessments for every patient. Consequently, many high-risk adolescents may remain undetected, highlighting the urgent need for objective, data-driven predictive models (17).

Recent advancements in AI-based depression recognition have shown promise in enhancing early diagnosis and suicide risk prediction (18–20). AI techniques, such as deep learning models and natural language processing, are increasingly applied to analyze large-scale behavioral or physiological data for timely identification of depressive symptoms (21, 22). However, these AI-driven systems face limitations, particularly when applied to adolescents in non-Western cultural contexts (23). Digital phenotypes of depression can vary significantly across cultures, and adolescents often display complex emotional expressions that challenge the generalizability of automated models (24, 25). Additionally, issues like limited data accessibility, device usage restrictions, and concerns about privacy and stigma further limit AI’s applicability (26, 27).

To address these limitations, this study adopts a data-driven predictive modeling approach that integrates Least Absolute Shrinkage and Selection Operator (Lasso) regression and logistic regression, both of which are highly effective for handling high-dimensional psychiatric data and improving prediction accuracy (G. E. 28–30). Lasso regression automatically selects the most relevant predictive factors, effectively managing multicollinearity and reducing overfitting, particularly in high-dimensional psychiatric datasets (31–34). Logistic regression, widely recognized for its statistical interpretability and clinical applicability, enables objective, data-driven decision-making and is commonly applied in mental health prediction research (35, 36). By combining these techniques, our model aims to improve predictive accuracy and optimize resource allocation, thereby facilitating early identification of high-risk adolescents in psychiatric inpatient settings (37).

Recent research has increasingly focused on developing predictive models for adolescent suicide risk, aiming to enhance prediction accuracy through the integration of psychosocial and behavioral factors. For instance, Walsh et al. (38) employed machine learning techniques, including random forests and support vector machines, to create a suicide risk prediction model for adolescents, achieving notable predictive accuracy (38). Furthermore, studies have identified emotional dysregulation, psychosocial stressors, and significant life events as critical contributors to suicidal ideation and behavior (39). Additionally, family and peer support has been shown to mitigate the adverse psychological impact of stress, thereby reinforcing the protective role of social networks in adolescent mental health (Y.-L. 40, 41).

Despite extensive research on adolescent suicide risk factors, most studies have employed case-control designs that primarily compare depressed adolescents to the general population, rather than identifying high-risk subgroups within clinical settings (6). However, emerging evidence suggests that adolescents diagnosed with depression exhibit distinct cognitive, emotional, and behavioral profiles compared to their non-depressed peers, highlighting the need for more specialized risk prediction approaches (42, 43). Generalized models that do not account for these subgroup-specific characteristics may lead to systematic misclassification of risk levels, ultimately limiting their practical utility in suicide prevention among hospitalized adolescents (44, 45).

A key limitation of existing suicide prediction models is their heavy reliance on data predominantly derived from Western populations, which may not adequately capture the sociocultural influences on suicidal behavior among Chinese adolescents (Y. 46). Research indicates that family dynamics, academic stress, and societal attitudes toward mental health differ significantly between China and Western contexts, potentially affecting the predictive validity of risk factors identified in Western-based models (S. 47, 48). Despite these well-documented cultural variations, few studies have systematically assessed whether these risk models retain their accuracy across diverse populations.

Addressing this gap necessitates the development of a suicide risk prediction model specifically tailored for hospitalized adolescents with depression in China. Such a model should integrate culturally relevant psychosocial factors alongside established clinical predictors, thereby enhancing its applicability in Chinese psychiatric settings. By addressing these limitations, this study aims to improve suicide risk stratification and support more effective early intervention strategies in clinical practice.

The present study aims to develop a suicide risk prediction model for hospitalized adolescents with depression in China. Specifically, we seek to identify key predictive factors using Lasso regression and logistic regression, and to evaluate the model’s predictive performance through internal validation, which includes the area under the curve (AUC), calibration curves, and decision curve analysis (DCA). Furthermore, we explore the potential clinical implications of this model, highlighting the need for future external validation studies to establish its broader applicability.

## 2 Materials and methods

### 2.1 Study participants

This retrospective study examined inpatient adolescents aged 13 to 18 years who were diagnosed with depression and admitted to the psychiatric ward of the First Hospital of Shanxi Medical University from June 2022 to June 2023. The inclusion criteria mandated that each participant receive a clinical diagnosis of depression confirmed by a psychiatrist in accordance with ICD-10 criteria. Patients with a history of depression attributable to organic diseases, such as epilepsy, traumatic brain injury, or neurodegenerative disorders, were excluded. Furthermore, individuals with severe mental disorders, including schizophrenia, schizoaffective disorder, bipolar disorder (manic or mixed episodes), or other psychotic disorders, as well as those with intellectual disabilities (IQ < 70), were not included to ensure their capacity to understand and complete the assessments. Patients whose psychological evaluations contained inconsistent responses were also excluded from the analysis.

To ensure analytical independence and model validity, we adhered to the Events Per Variable (EPV) principle, establishing a standard of 10 EPV. With an anticipated selection of 5 to 10 predictor variables, a minimum sample size of 100 to 150 cases was necessary. The final sample of 229 patients met these criteria, facilitating robust predictor identification through Lasso regression. Although sufficient for initial model development, future studies should aim for larger, multicenter cohorts to enhance external validation and generalizability.

Initially, 232 participants were assessed; however, three were excluded due to inconsistent responses, resulting in a final cohort of 229 adolescents.

This study was conducted in accordance with the Declaration of Helsinki and received approval from the Ethics Committee of the First Hospital of Shanxi Medical University (Approval No. 21k-149). Written informed consent was obtained from all participants and their legal guardians, along with institutional approval from the hospital authorities.

### 2.2 Data collection procedures

Data were collected prospectively by experienced psychiatric nurses, each possessing a minimum of five years of clinical experience in psychiatric care. Prior to data collection, all nurses underwent structured training to ensure the consistent administration of assessments and adherence to standardized protocols. Assessments were conducted face-to-face in a designated private room within the psychiatric ward, thereby ensuring confidentiality and minimizing external distractions. Given the comprehensive nature of the study, which included one demographic questionnaire and 15 validated self-report scales, the data collection process necessitated a prolonged completion time. To mitigate respondent fatigue, assessments were conducted within the first week of hospitalization, allowing participants to take breaks as needed.

All instruments in this study were self-administered, with psychiatric nurses providing clarification only when participants experienced difficulty understanding specific items. Upon completion, all questionnaires were immediately reviewed for completeness and logical consistency. If any missing or ambiguous responses were identified, nurses sought clarification before finalizing the dataset. These measures ensured high data reliability and minimized bias in self-reported responses.

The entire data collection period spanned approximately one year, from June 2022 to June 2023.

### 2.3 Study tools

This study employed a range of validated tools and scales to collect data on participants’ demographics, individual characteristics, family environment, social support, and mental health. All instruments used in this study were validated scales with established psychometric properties specifically tailored for adolescent populations. Most of these tools have been previously adapted and validated for use among Chinese adolescents, demonstrating appropriate reliability and applicability within this cultural context. For instance, the Self-Rating Depression Scale (SDS) has been extensively utilized in China, with studies confirming its internal consistency (Cronbach’s α = 0.847) among samples of Chinese adolescents. Similarly, the Pittsburgh Sleep Quality Index (PSQI) has been validated for Chinese populations, ensuring its relevance in assessing sleep disturbances within the context of this study. By confirming that all instruments possess psychometric soundness within the study population, we aimed to enhance the reliability and validity of our findings. The Cronbach’s alpha coefficients for these instruments were all above 0.7, demonstrating good internal consistency.

#### 2.3.1 Demographic data

Demographic data were collected, encompassing essential information such as age, gender, religious beliefs, family background (including the number of siblings, family economic status, and family history of depression), education level, and area of residence. Additionally, factors of adversity experienced by participants were documented, including histories of trauma, school bullying, and instances of domestic violence.

#### 2.3.2 Psychological and social assessment tools

This study employed a range of standardized assessment scales to conduct a comprehensive evaluation of psychological, social, and familial variables. The tools utilized included:

The Self-rating Depression Scale (SDS) is a 20-item self-report instrument developed by Zung to assess the presence and severity of depressive symptoms. his scale is widely used in both clinical and research settings to screen for depression and monitor treatment response. In this study, the Cronbach’s alpha was 0.847, indicating good internal consistency (49).

The Self-Injury Questionnaire for Youth (SHQ-At): The SHQ-At assesses the frequency, methods, and functions of non-suicidal self-injury (NSSI) in adolescents. It includes items on triggers and emotional regulation related to self-harm. Higher scores indicate greater engagement in NSSI. In this study, Cronbach’s alpha = 0.921 (50).

The Short Form Coping Style Questionnaire (SCSQ): The SCSQ measures coping strategies in response to stress, divided into Active Coping (e.g., problem-solving) and Negative Coping (e.g., avoidance). Higher scores reflect greater reliance on specific coping styles, with a reported Cronbach’s alpha of 0.824 in this study (51, 52).

The Adolescent Life Events Scale (ASLEC): The ASLEC assesses stressful life events in adolescents across six domains: Interpersonal Relationships, Study Pressure, Punishment, Loss, Health Adaptation, and Other. Higher scores indicate greater exposure to negative life events, with a Cronbach’s alpha of 0.894 in this study (53).

The Dual System Scale of Self-Control for Adolescents (DMSC-SA): This is a tool designed to assess the self-control abilities of adolescents. In this study, the Cronbach’s alpha value for the impulse system is 0.849,while the control system has a Cronbach’s alpha of 0.831 (54).

The Dysfunctional Attitudes Scale (DAS): The DAS evaluates maladaptive cognitive patterns related to depression, including perfectionism, need for approval, dependency, and autonomy attitudes. Higher scores indicate more dysfunctional beliefs. Cronbach’s alpha = 0.906 in this study (55).

The General Self-Efficacy Scale (GSES): The GSES measures an individual’s perceived ability to cope with challenges and achieve goals, with higher scores reflecting greater self-efficacy. Cronbach’s alpha = 0.864 in this study (56).

The Resilience Scale (CD-RISC): The CD-RISC assesses psychological resilience, including stress coping ability, emotional regulation, and adaptability. Higher scores indicate greater resilience. Cronbach’s alpha = 0.907 in this study (57, 58).

The Pittsburgh Sleep Quality Index (PSQI): The PSQI evaluates subjective sleep quality, covering sleep duration, latency, efficiency, disturbances, and daytime dysfunction. Higher scores indicate poorer sleep quality. Cronbach’s alpha = 0.816 in this study (59).

The Multidimensional Fatigue Inventory (MFI-20): The MFI-20 assesses fatigue levels across five dimensions: general fatigue, physical fatigue, mental fatigue, reduced activity, and reduced motivation. Higher scores indicate greater fatigue. Cronbach’s alpha = 0.864 in this study (60).

The Link Stigma Scale Series (LSSS): The LSSS evaluates stigma perceptions related to mental health, including experiences of discrimination, perceived societal attitudes, and self-stigmatization. Higher scores indicate greater perceived stigma, with a Cronbach’s alpha of 0.858 in this study (61).

The Parenting Style Questionnaire (PBI): The PBI assesses parenting styles across two key dimensions: parental care (warmth and emotional support) and parental overprotection (control and intrusion). Higher scores on the care dimension reflect greater parental warmth, whereas higher scores on overprotection suggest more controlling behaviors. In this study, the Cronbach’s alpha was found to be 0.934 (62).

The Family Functioning Assessment Scale (FAD): The FAD evaluates family functioning across multiple domains, including problem-solving, communication, roles, affective involvement, and behavior control. Higher scores indicate greater family dysfunction. The Cronbach’s alpha for this study was 0.875 (63).

The Family Closeness and Adaptability Scale (Chinese version) (FACESII-CV): This scale evaluates family closeness and adaptability, with a Cronbach’s alpha of 0.929(X.-Y. 64).

The Perceived School Climate Scale (PSCS): The PSCS measures students’ perceptions of school climate, focusing on three dimensions: teacher support, peer support, and autonomy in learning. Higher scores indicate a more positive and supportive school environment, which is associated with better academic engagement and emotional well-being. The Cronbach’s alpha in this study was 0.794 (65).

### 2.4 Data analysis

All statistical analyses were conducted using IBM SPSS version 25.0 and R version 4.1.2.

Categorical variables were reported as frequencies (%) and analyzed using the χ² test. Continuous variables were assessed for normality through the Shapiro-Wilk test, skewness and kurtosis Z-scores (with a threshold of ±1.96), and visual inspection methods, including Q-Q plots and boxplots. Although the skewness and kurtosis Z-scores indicated that most variables were approximately normally distributed (see **Supplementary Table S1**), we adopted a more conservative criterion for robust statistical analysis. Variables exhibiting borderline or minor deviations from normality, as determined by any of these assessments, were analyzed using non-parametric methods (Mann-Whitney U test) and reported as median (P25, P75). Only those variables that clearly met the normality criteria across all assessments were presented as mean ± standard deviation and compared using t-tests.

To identify significant predictor variables, Lasso regression was initially employed. This technique, which utilizes L1 regularization, effectively selects key variables associated with suicide risk from high-dimensional data while reducing redundancy and minimizing overfitting. The optimalλparameter was determined using 10-fold cross-validation, with the selection criterion based on minimizing the mean squared error to enhance model stability. Given the broad range of social, psychological, and familial factors included in this study, Lasso regression played a crucial role in identifying the most relevant features, thereby improving both model accuracy and generalizability.

The significant predictor variables identified through Lasso regression were subsequently incorporated into a multivariate logistic regression model to assess their association with suicide risk. The final model was selected using a bidirectional stepwise approach, optimizing both predictive performance and parsimony based on the Akaike Information Criterion (AIC).

To thoroughly evaluate the model’s performance, we assessed its discrimination, calibration, and clinical applicability. Receiver Operating Characteristic (ROC) curves were generated to determine the model’s capacity to distinguish between individuals with and without suicide risk, with AUC serving as an indicator of overall predictive accuracy. Sensitivity and specificity were utilized to measure the model’s ability to identify individuals at risk for suicide and to exclude those who are not at risk, respectively. The Hosmer-Lemeshow test was employed to evaluate the model’s goodness-of-fit, and calibration curves were used to further validate the agreement between predicted and observed risks.

Additionally, the clinical utility of the model was evaluated using DCA and Clinical Impact Curves (CIC), providing insights into its potential benefits across various clinical decision-making scenarios. A statistical significance threshold of *P* < 0.05 was established for all analyses.

## 3 Results

### 3.1 Baseline characteristics

**Figure 1:**
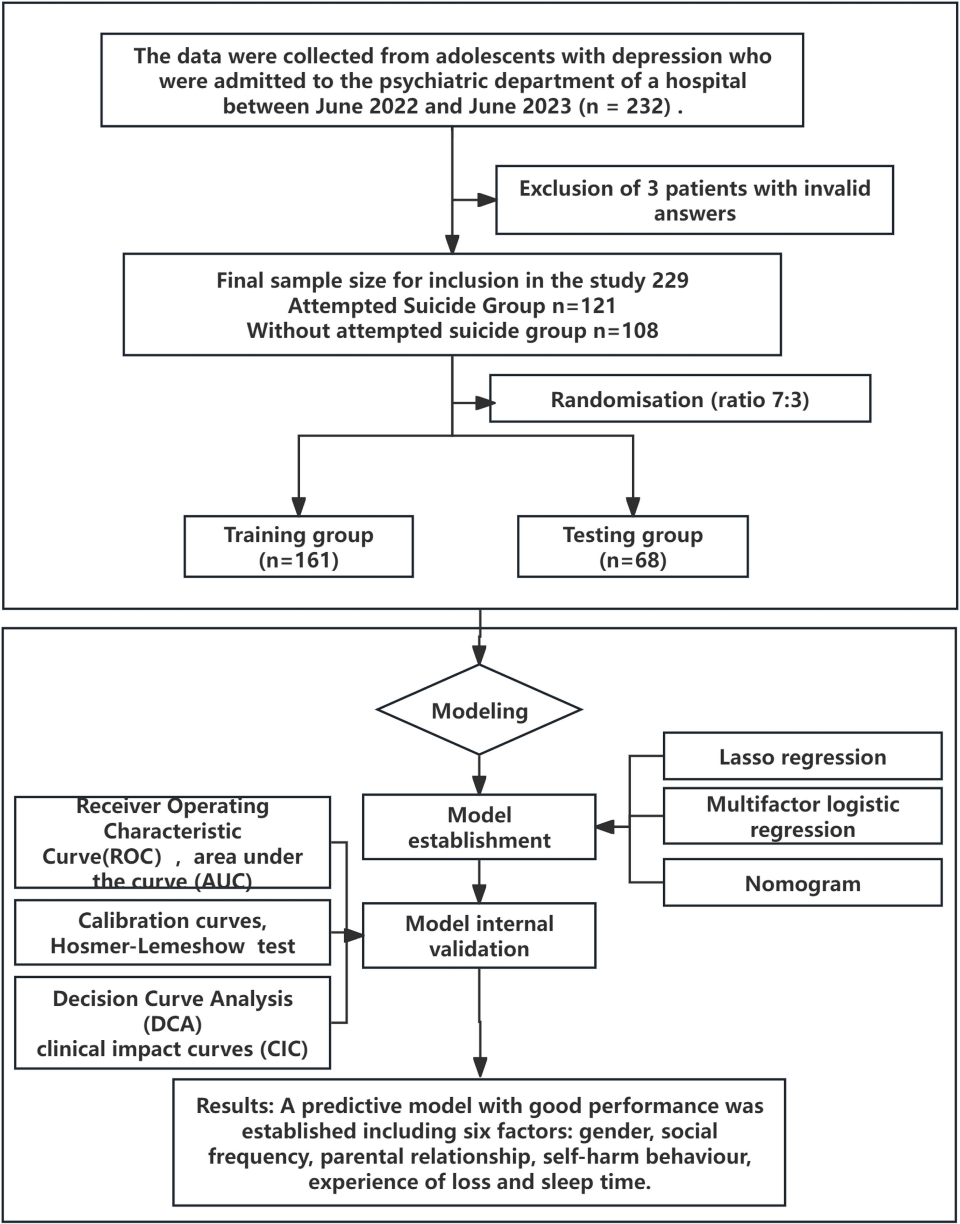
Research flowchart.

This study involved 229 patients, of whom 121 had a history of suicide attempts and 108 did not. Each participant completed a general demographic questionnaire and 15 validated psychological scales, resulting in a total of 3664 individual survey responses. Participants were randomly assigned to a training set (n = 161, 70.3%) and a testing set (n = 68, 29.7%), as illustrated in **Figure 1**. The testing set was used exclusively for final model evaluation and was not involved in model selection or parameter tuning.

Baseline characteristics of the study population are summarized in **Table 1**. No significant differences were observed between the training and testing sets for demographic variables (e.g., gender, age, place of residence, and education level) or psychological characteristics (e.g., depression severity, coping styles, and history of traumatic experiences). The majority of baseline characteristics were well-balanced, with the exception of the LSSS shame dimension, the adaptability dimension of the FACESII-CV, and the sleep disorder transformation dimension of the PSQI, which showed statistically significant differences between groups. However, these variables were not included in the subsequent predictor selection process.

**Table 1:** Baseline characteristics of the study population.

Variables	Total (n = 229)	Training set	Testing set	P
M (Q_1_, Q_3_)		(n = 161)	(n = 68)	
grouped, n(%):				0.901
No suicide attempts	108 (47.16%)	75 (46.58%)	33 (48.53%)	
Suicide Attempt	121 (52.84%)	86 (53.42%)	35 (51.47%)	
Gender, n(%):				0.390
Male	82 (35.81%)	61 (37.89%)	21 (30.88%)	
Female	147 (64.19%)	100 (62.11%)	47 (69.12%)	
Ethnic, n(%):				0.507
None	227 (99.13%)	160 (99.38%)	67 (98.53%)	
Yes	2 (0.87%)	1 (0.62%)	1 (1.47%)	
Religion, n(%):				1.000
None	221 (96.51%)	66 (97.06%)	155 (96.27%)	
Yes	8 (3.49%)	2 (2.94%)	6 (3.73%)	
Siblings, n(%):				0.922
None	68 (29.69%)	47 (29.19%)	21 (30.88%)	
Yes	161 (70.31%)	114 (70.81%)	47 (69.12%)	
Place of residence, n(%):				0.236
Urban	190 (82.97%)	130 (80.75%)	60 (88.24%)	
Rural	39 (17.03%)	31 (19.25%)	8 (11.76%)	
School district, n(%):				0.159
Urban	213 (93.01%)	147 (91.30%)	66 (97.06%)	
Rural	16 (6.99%)	14 (8.70%)	2 (2.94%)	
Level of education, n(%)	3.00 [2.00;3.00]	3.00 [2.00;3.00]	3.00 [2.00;3.00]	0.081
Junior high school	76 (33.19)	26 (24.07)	50 (41.32)	
Senior high school or technical school	128 (55.90)	67 (62.04)	61 (50.41)	
College/University and above in school	9 (3.93)	6 (5.56)	3 (2.48)	
Dropped out	10 (4.37)	6 (5.56)	4 (3.31)	
Graduated	6 (2.62)	3 (2.78)	3 (2.48)	
Grades, n(%)				0.939
Excellent	32 (13.97%)	23 (14.29%)	9 (13.24%)	
Above average	64 (27.95%)	46 (28.57%)	18 (26.47%)	
Average	76 (33.19%)	51 (31.68%)	25 (36.76%)	
Below average	40 (17.47%)	28 (17.39%)	12 (17.65%)	
Poor	17 (7.42%)	13 (8.07%)	4 (5.88%)	
History of school violence, n(%)				0.228
None	119 (51.97%)	79 (49.07%)	40 (58.82%)	
Yes	110 (48.03%)	82 (50.93%)	28 (41.18%)	
Traumatic experiences, n(%)				0.085
None	130 (56.77%)	85 (52.80%)	45 (66.18%)	
Yes	99 (43.23%)	76 (47.20%)	23 (33.82%)	
Love experience, n(%)				1.000
None	166 (72.49%)	117 (72.67%)	49 (72.06%)	
Yes	63 (27.51%)	44 (27.33%)	19 (27.94%)	
domestic violence, n(%)				0.500
None	132 (57.64%)	90 (55.90%)	42 (61.76%)	
Yes	97 (42.36%)	71 (44.10%)	26 (38.24%)	
Whether parents live or not, n(%)				1.000
None	225 (98.25%)	158 (98.14%)	67 (98.53%)	
Yes	4 (1.75%)	3 (1.86%)	1 (1.47%)	
Parental marital status, n(%)				0.747
None	23 (10.04%)	15 (9.32%)	8 (11.76%)	
Yes	206 (89.96%)	146 (90.68%)	60 (88.24%)	
Parental relationships, n(%)				0.416
Harmonious	25 (10.92%)	15 (9.32%)	10 (14.71%)	
Occasional conflicts	61 (26.64%)	43 (26.71%)	18 (26.47%)	
Frequent conflicts	78 (34.06%)	53 (32.92%)	25 (36.76%)	
Separated/divorced	65 (28.38%)	50 (31.06%)	15 (22.06%)	
Living situation, n(%)				0.766
living with parents	170 (74.24%)	120 (74.53%)	50 (73.53%)	
Living with grandparents	16 (6.99%)	12 (7.45%)	4 (5.88%)	
Living with father	2 (0.87%)	2 (1.24%)	0 (0.00%)	
Living with mother	31 (13.54%)	19 (11.80%)	12 (17.65%)	
Living alone	3 (1.31%)	3 (1.86%)	0 (0.00%)	
Other	7 (3.06%)	5 (3.11%)	2 (2.94%)	
Immediate family history of depression, n(%)				0.253
None	198 (86.46%)	136 (84.47%)	62 (91.18%)	
Yes	31 (13.54%)	25 (15.53%)	6 (8.82%)	
Immediate family history of other mental illness, n(%)				0.354
None	216 (94.32%)	150 (93.17%)	66 (97.06%)	
Yes	13 (5.68%)	11 (6.83%)	2 (2.94%)	
Household income, n(%)				0.105
Below 2000 RMB	24 (10.48)	16 (14.81)	8 (6.61)	
2000–4000 RMB	34 (14.85)	11 (10.19)	23 (19.01)	
4000–6000 RMB	82 (35.81)	42 (38.89)	40 (33.06)	
6000-10,000 RMB	49 (21.40)	22 (20.37)	27 (22.31)	
Above 10,000 RMB	40 (17.47)	17 (15.74)	23 (19.01)	
Household financial situation, n(%)				0.645
Good	33 (14.41%)	23 (14.29%)	10 (14.71%)	
Average	163 (71.18%)	117 (72.67%)	46 (67.65%)	
Poor	33 (14.41%)	21 (13.04%)	12 (17.65%)	
Father’s culture, n(%)				0.135
Primary school or below	30 (13.10)	13 (12.04)	17 (14.05)	
Junior high school	72 (31.44)	30 (27.78)	42 (34.71)	
Senior high school or technical school	51 (22.27)	24 (22.22)	27 (22.31)	
College/university	71 (31.00)	36 (33.33)	35 (28.93)	
Master’s degree or above	5 (2.18)	5 (4.63)	0 (0.00)	
Mother’s culture, n(%)				0.794
Primary school or below	26 (11.35)	14 (12.96)	12 (9.92)	
Junior high school	88 (38.43)	45 (41.67)	43 (35.54)	
Senior high school or technical school	40 (17.47)	11 (10.19)	29 (23.97)	
College/university	69 (30.13)	38 (35.19)	31 (25.62)	
Master’s degree or above	6 (2.62)	0 (0.00)	6 (4.96)	
Father’s occupation, n(%)				0.329
Mental work	83 (36.24%)	60 (37.27%)	23 (33.82%)	
Physical labor	96 (41.92%)	69 (42.86%)	27 (39.71%)	
Freelancer	39 (17.03%)	27 (16.77%)	12 (17.65%)	
Unemployed	11 (4.80%)	5 (3.11%)	6 (8.82%)	
Mother’s occupation, n(%)				0.868
Mental work	82 (35.81%)	57 (35.40%)	25 (36.76%)	
Physical labor	39 (17.03%)	26 (16.15%)	13 (19.12%)	
Freelancer	39 (17.03%)	27 (16.77%)	12 (17.65%)	
Unemployed	69 (30.13%)	51 (31.68%)	18 (26.47%)	
Social frequency, n(%)				0.359
Almost every day	21 (9.17%)	13 (8.07%)	8 (11.76%)	
1–3 days 3	25 (10.92%)	17 (10.56%)	8 (11.76%)	
3–7 days	46 (20.09%)	37 (22.98%)	9 (13.24%)	
1–2 weeks	137 (59.83%)	94 (58.39%)	43 (63.24%)	
Close friends, n(%)	4.00 [3.00;5.00]	4.00 [3.00;5.00]	4.00 [3.00;5.00]	0.078
Many	5 (2.18)	0 (0.00)	5 (4.13)	
Quite a few	26 (11.35)	17 (15.74)	9 (7.44)	
Average	42 (18.34)	18 (16.67)	24 (19.83)	
few	51 (22.27)	25 (23.15)	26 (21.49)	
Very few	105 (45.85)	48 (44.44)	57 (47.11)	
Chronic illness history, n(%)				0.142
None	203 (88.65%)	139 (86.34%)	64 (94.12%)	
Yes	26 (11.35%)	22 (13.66%)	4 (5.88%)	
HEIGHT	167.00 [163.00;173.00]	167.00 [163.00;172.00]	167.00 [163.00;173.25]	0.798
BMI	21.00 [19.00;24.00]	21.00 [18.00;24.00]	21.00 [19.00;24.00]	0.839
AGE	15.00 [14.00;16.00]	15.00 [14.00;16.00]	15.00 [13.00;16.00]	0.543
SHQAt	22.00 [10.00;34.00]	21.00 [10.00;33.00]	24.00 [13.75;35.25]	0.28
SCSQ.AR	12.00 [8.00;17.00]	13.00 [8.00;17.00]	11.50 [8.00;16.25]	0.369
SCSQ.NC, Mean ± SD	11.33 (4.90)	11.22 (4.87)	11.59 (5.02)	0.613
SDS	59.00 [54.00;68.00]	58.00 [54.00;68.00]	59.00 [53.75;65.75]	0.946
ASLEC.IR	12.00 [7.00;16.00]	12.00 [7.00;16.00]	12.50 [8.00;17.00]	0.535
ASLEC.SP	11.00 [6.00;15.00]	10.00 [6.00;15.00]	12.00 [7.75;16.00]	0.162
ASLEC.P	9.00 [4.00;15.00]	9.00 [4.00;15.00]	10.00 [4.75;14.50]	0.371
ASLEC.F	1.00 [0.00;4.00]	2.00 [0.00;4.00]	1.00 [0.00;5.00]	0.91
ASLEC.HA	4.00 [2.00;7.00]	4.00 [2.00;7.00]	4.50 [2.00;7.00]	0.47
ASLEC.O	7.00 [5.00;9.00]	7.00 [4.00;9.00]	7.00 [5.00;10.00]	0.453
LSSS.DDS	2.50 [2.25;2.75]	2.50 [2.25;2.75]	2.50 [2.25;2.75]	0.968
LSSS.SPCS.P	2.22 [2.00;2.67]	2.22 [2.00;2.67]	2.22 [2.00;2.67]	0.773
LSSS.SPCS.F	2.25 [1.88;2.50]	2.25 [2.00;2.50]	2.19 [1.63;2.38]	0.017
LSSS.SPCS.E	2.00 [2.00;2.67]	2.33 [2.00;2.67]	2.00 [1.67;2.42]	0.113
LSSS.SPCS.C	2.00 [1.50;2.25]	2.00 [1.75;2.25]	2.00 [1.25;2.25]	0.553
LSSS.SPCS.SE	2.33 [2.00;3.00]	2.33 [2.00;3.00]	2.33 [2.00;3.00]	0.703
LSSS.SAES.M	2.00 [1.33;2.00]	2.00 [1.33;2.00]	2.00 [1.33;2.08]	0.942
LSSS.SAES.DS	2.50 [2.00;2.75]	2.25 [2.00;2.75]	2.50 [2.00;3.00]	0.119
GSES	18.00 [14.00;21.00]	18.00 [15.00;21.00]	17.50 [14.00;21.00]	0.772
DMSC.SA.ISI	17.00 [12.00;22.00]	17.00 [12.00;21.00]	17.50 [12.00;22.00]	0.796
DMSC.SA.ISD	12.00 [9.00;13.00]	11.00 [9.00;13.00]	12.00 [9.75;13.00]	0.529
DMSC.SA.ISDG	8.00 [6.00;10.00]	8.00 [6.00;10.00]	8.00 [6.00;10.00]	0.619
DMSC.SA.CSPS	20.00 [18.00;23.00]	20.00 [18.00;23.00]	21.00 [17.00;23.00]	0.743
DMSC.SA.CSFT	7.00 [4.00;9.00]	7.00 [5.00;9.00]	6.00 [3.00;8.00]	0.052
DAS.V, Mean ± SD	21.49 (5.13)	21.12 (5.13)	22.35 (5.06)	0.097
DAS.AR	23.00 [20.00;27.00]	23.00 [20.00;26.00]	24.00 [21.00;27.00]	0.242
DAS.P	23.00 [18.00;28.00]	23.00 [18.00;27.00]	24.50 [20.00;28.00]	0.283
DAS.M, Mean ± SD	20.49 (5.08)	20.80 (5.20)	19.78 (4.74)	0.152
DAS.AT, Mean ± SD	21.87 (5.70)	21.75 (6.04)	22.16 (4.84)	0.588
DAS.D, Mean ± SD	21.53 (5.10)	21.48 (5.18)	21.65 (4.93)	0.823
DAS.AA	24.00 [19.00;30.00]	24.00 [18.00;30.00]	25.50 [21.75;30.00]	0.149
DAS.CP, Mean ± SD	19.77 (5.57)	20.22 (5.44)	18.72 (5.79)	0.071
CD.RISC.T	16.00 [11.00;22.00]	17.00 [11.00;23.00]	14.50 [10.00;21.00]	0.247
CD.RISC.C	12.00 [9.00;15.00]	12.00 [9.00;16.00]	12.00 [9.00;14.00]	0.31
CD.RISC.O	6.00 [4.00;8.00]	6.00 [4.00;8.00]	6.00 [4.00;8.00]	0.765
FACESII.CV.I, Mean ± SD	57.27 (12.08)	58.12 (11.64)	55.26 (12.93)	0.119
FACESII.CV.A	37.00 [32.00;45.00]	39.00 [32.00;47.00]	34.00 [29.75;43.00]	0.015
MFI.20.GF	13.00 [12.00;15.00]	13.00 [12.00;15.00]	13.00 [12.00;16.00]	0.788
MFI.20.PF	11.00 [10.00;13.00]	12.00 [10.00;14.00]	11.00 [9.00;13.00]	0.243
MFI.20.MF	12.00 [11.00;15.00]	12.00 [11.00;15.00]	12.00 [11.00;14.00]	0.86
MFI.20.RA	12.00 [10.00;14.00]	12.00 [10.00;14.00]	12.00 [10.00;14.25]	0.653
MFI.20.DM	13.00 [11.00;15.00]	13.00 [11.00;15.00]	14.00 [11.00;14.25]	0.524
PBI.I.F	42.00 [35.00;49.00]	42.00 [35.00;50.00]	41.50 [33.75;47.25]	0.395
PBI.I.M	45.00 [39.00;54.00]	45.00 [39.00;55.00]	45.50 [38.75;53.25]	0.742
PBI.II.F	21.00 [16.00;28.00]	20.00 [16.00;27.00]	23.00 [17.00;28.75]	0.106
PBI.II.M	39.00 [33.00;44.00]	38.00 [33.00;44.00]	40.50 [34.00;45.25]	0.155
PBI.III.F	22.00 [20.00;27.00]	22.00 [20.00;27.00]	22.00 [19.75;27.00]	0.845
PBI.III.M	16.00 [12.00;20.00]	16.00 [12.00;19.00]	17.00 [13.00;20.00]	0.350
PBI.IV.F	9.00 [7.00;12.00]	9.00 [7.00;12.00]	9.00 [5.00;11.25]	0.088
PBI.IV.M	16.00 [11.00;21.00]	17.00 [11.00;20.00]	15.50 [11.75;21.00]	0.685
PBI.V.F	11.00 [9.00;14.00]	11.00 [8.00;14.00]	12.00 [9.75;14.00]	0.127
PBI.V.M	9.00 [7.00;13.00]	10.00 [7.00;13.00]	9.00 [6.00;12.00]	0.279
PBI.VI.F	11.00 [9.00;13.00]	11.00 [9.00;13.00]	11.00 [9.75;13.25]	0.437
FAD.PS	14.00 [12.00;16.00]	14.00 [12.00;16.00]	15.00 [13.00;16.00]	0.112
FAD.C	22.00 [21.00;24.00]	22.00 [20.00;24.00]	22.00 [21.00;23.00]	0.516
FAD.R	27.00 [25.00;29.00]	27.00 [25.00;29.00]	27.50 [24.00;30.00]	0.888
FAD.ER	15.00 [13.00;16.00]	15.00 [13.00;16.00]	14.00 [12.00;16.00]	0.323
FAD.EI	17.00 [15.00;19.00]	17.00 [15.00;19.00]	17.00 [14.00;19.00]	0.475
FAD.BC	23.00 [21.00;25.00]	23.00 [21.00;25.00]	23.00 [21.00;24.00]	0.996
FAD.TF	28.00 [26.00;31.00]	29.00 [26.00;31.00]	28.00 [27.00;32.00]	0.576
PSQI A	1.00 [1.00, 2.00]	1.00 [1.00, 2.00]	1.00 [1.00, 2.00]	0.272
PSQI B	2.00 [2.00, 3.00]	2.00 [2.00, 3.00]	2.00 [2.00, 3.00]	0.402
PSQI C	1.00 [0.00, 2.00]	0.50 [0.00, 2.00]	1.00 [0.00, 2.00]	0.190
PSQI D	0.00 [0.00, 1.00]	0.00 [0.00, 1.00]	0.00 [0.00, 1.00]	0.132
PSQI E	2.00 [1.00, 2.00]	1.00 [1.00, 2.00]	2.00 [1.00, 2.00]	0.013
PSQI F	1.00 [0.00, 2.00]	0.00 [0.00, 2.00]	1.00 [0.00, 2.00]	0.058
PSQI G	2.00 [1.00, 2.00]	2.00 [1.00, 2.00]	2.00 [1.00, 3.00]	0.430
PSCS.T	16.00 [13.00;20.00]	16.00 [13.00;20.00]	16.00 [12.00;19.25]	0.334
PSCS.P	30.00 [27.00;33.00]	30.00 [27.00;33.00]	30.00 [27.00;32.00]	0.715
PSCS.A	10.00 [7.00;12.00]	10.00 [7.00;13.00]	10.00 [7.00;12.00]	0.277

a Mean ± SD: Mean and standard deviation; n(%): sample size and percentage.

b M: Median, Q_1_: 1st Quartile, Q_3_: 3rd Quartile.

c SCSQ.AR and SCSQ.NC represent the two dimensions of the SCSQ: Active Response and Negative Coping, respectively.

d ASLEC.IR, ASLEC.SP, ASLEC.P, ASLEC.F, ASLEC.HA, and ASLEC.O are the six dimensions of the ASLEC, corresponding to the Interpersonal Relationship factor, Study Pressure factor, Punishment factor, Forfeit factor, Healthy Adaptation factor, and Other factor.

e DMSC.SA.ISI, DMSC.SA.ISD, DMSC.SA.ISDG, and DMSC.SA.CSS represent the dimensions of the DMSC.SA: Impulse System (Impulsivity, Easy to Get Distracted, Delayed Gratification) and Control System (Problem Solving, Future Time Orientation).

f DAS.V, DAS.AR, DAS.P, DAS.M, DAS.AT, DAS.D, DAS.AA, and DAS.CP represent the eight dimensions of the DAS: Vulnerability, Attraction and Repulsion, Perfection, Mandatory, Need for Approval, Dependence, Autonomy Attitude, and Cognitive Philosophy.

g LSSS.DDS, LSSS.SPPS.P, LSSS.SPCS.F, LSSS.SPCS.E, LSSS.SPCS.C, and LSSS.SPCS.SE are the subscales of the LSSS: Disparaging Discrimination Scale (Disparagement) and Stigma Perception Pair Scale (Secrecy/Privacy, Flinch, Education, Challenge, Separate).

h CD.RISC.T, CD.RISC.C, and CD.RISC.O represent the three dimensions of the CD.RISC: Toughness, Competence, and Optimism.

i PSQI.A, PSQI.B, PSQI.C, PSQI.D, PSQI.E, PSQI.F, and PSQI.G are the seven dimensions of the PSQI: Sleep Quality, Sleep Onset, Sleep Duration, Sleep Efficiency, Sleep Disturbances, Hypnotic Medication, and Daytime Dysfunction.

j MFI.20.GF, MFI.20.PF, MFI.20.MF, MFI.20.RA, and MFI.20.DM are the five dimensions of the MFI.20: General Fatigue, Physical Fatigue, Mental Fatigue, Reduced Activity, and Decreased Motivation.

k PBI.I.F, PBI.II.M, PBI.III.F, PBI.IV.M, PBI.V.F, and PBI.VI.F are the factors of the PBI, measuring various parenting styles.

l FAD.PS, FAD.C, FAD.R, FAD.ER, FAD.EI, FAD.BC, and FAD.TF are the seven dimensions of the FAD: Problem Solving, Communication, Roles, Emotional Responses, Emotional Involvement, Behavioral Control, and Total Features.

m FACESII.CV.I and FACESII.CV.A represent the two dimensions of the FACESII.CV: Intimacy and Adaptability.

n PSCS.T, PSCS.P, and PSCS.A are the three dimensions of the PSCS: Teacher Support, Peer Support, and Autonomy Orientation.

### 3.2 Selection of predictor variables

**Figure 2:**
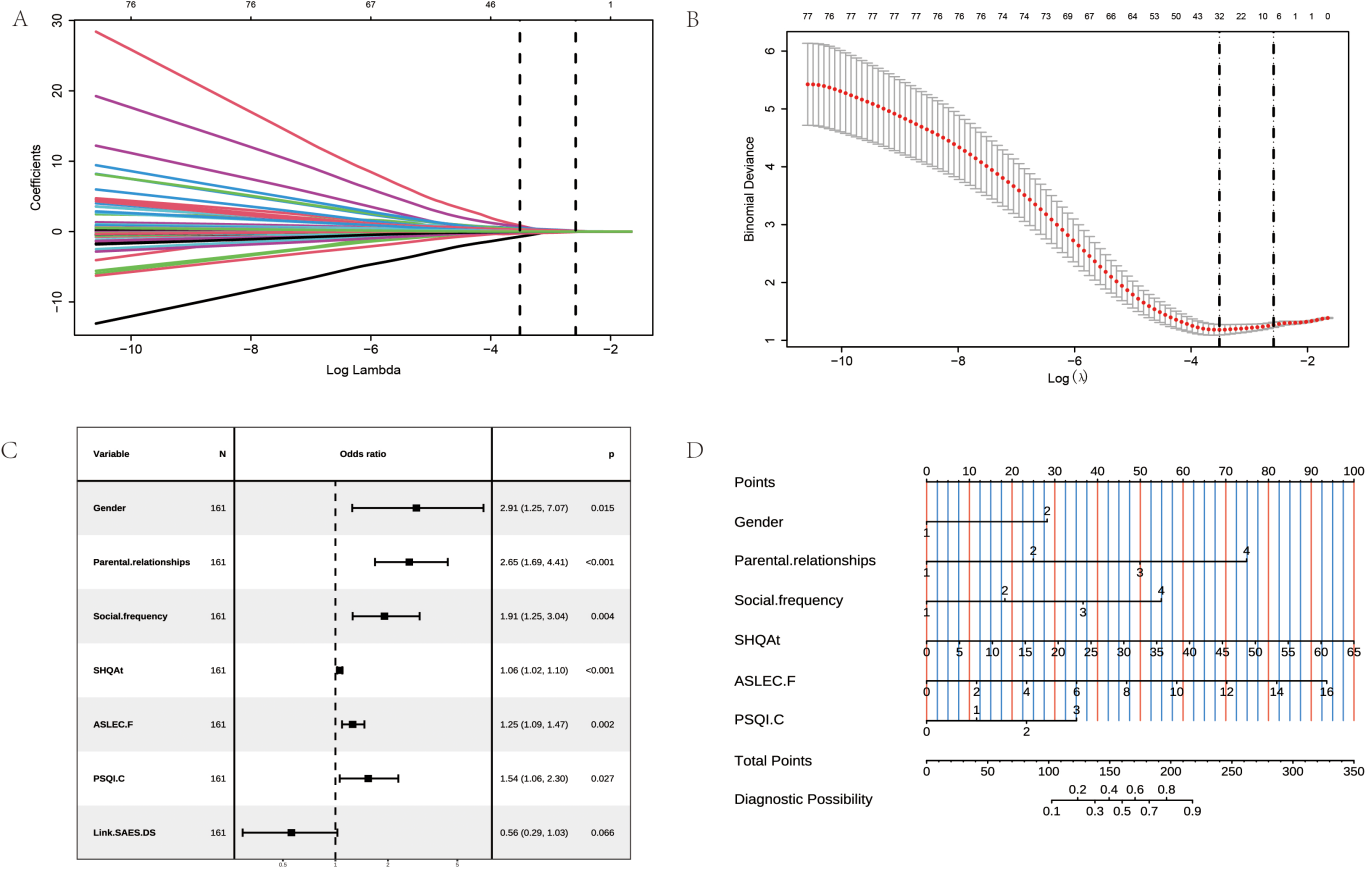
LASSO Regression and Nomogram. **(A)** Coefficient curve of the independent variables. **(B)** Optimal independent variable selection through LASSO regression with 10-fold cross-validation. **(C)** Multivariate logistic regression forest plot. **(D)** Nomogram for predicting suicide risk in adolescent depression.

Using the history of suicide attempts as the dependent variable, candidate predictors were screened through Lasso regression after excluding variables that exhibited baseline imbalances (see **Figures 2A, B**). To enhance model simplicity and clinical applicability, the optimal tuning parameter, lambda.1se (λ=0.076), was selected, resulting in the identification of seven significant predictor variables: gender, parental relationship, social frequency, self-harm behavior, the loss dimension of the ASLEC, the shame dimension of the LSSS, and the sleep duration dimension of the PSQI (see **Figure 2C**). Subsequently, these seven variables underwent multivariate logistic regression analysis, which indicated that six variables—gender, parental relationship, social frequency, self-harm behavior, loss dimension, and sleep duration—were significantly associated with suicidal tendencies (*P < 0.05*).

### 3.3 Model construction and performance evaluation

Based on multivariate logistic regression, the final model was selected utilizing AIC optimization, and a nomogram was constructed to visually represent the model’s predictive framework (**Figure 2D**). The total score for each individual was calculated from their predictor values, facilitating the estimation of their suicide risk probability.

**Figure 3:**
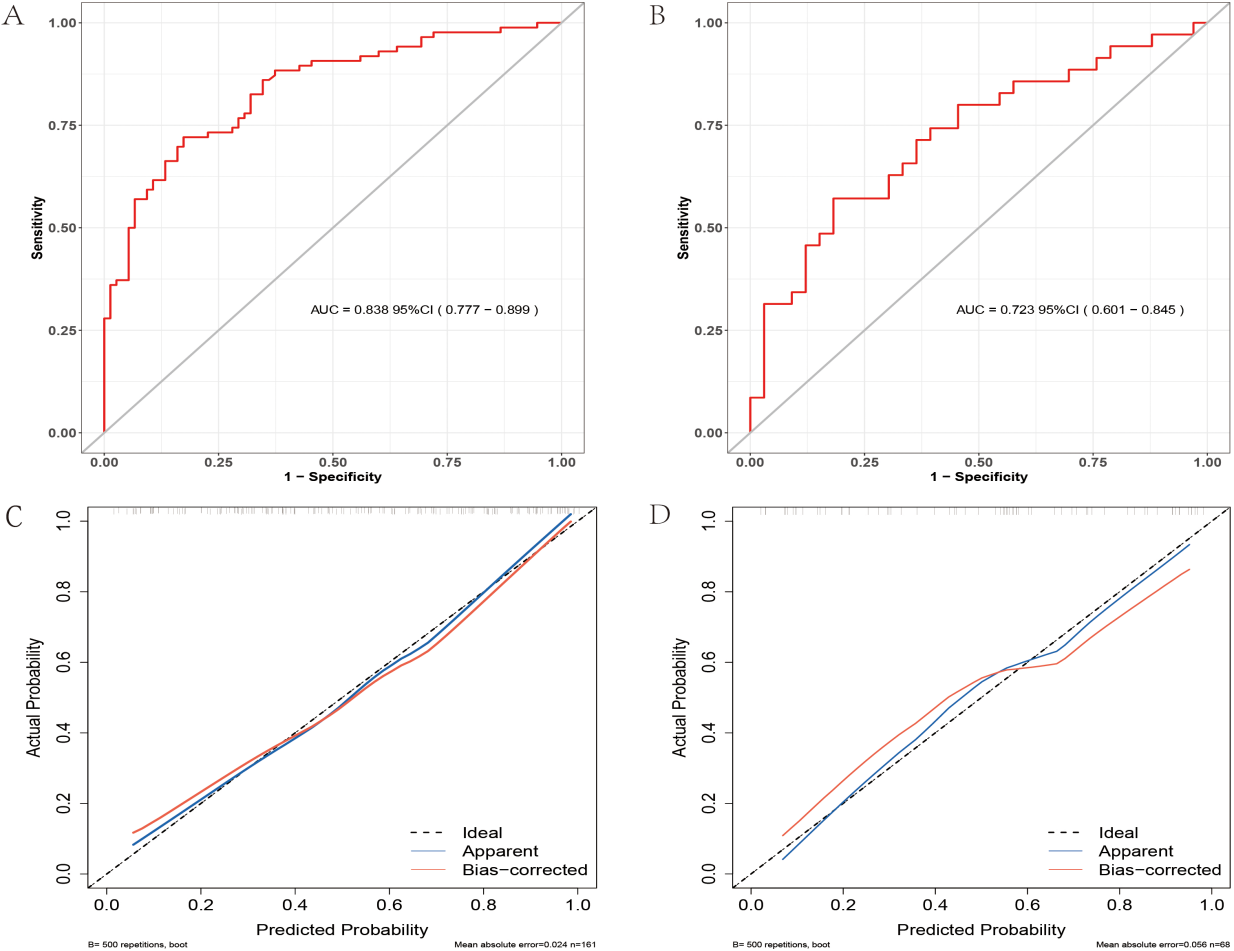
ROC and Calibration Curves. **(A)** ROC curve for the training group. **(B)** ROC curve for the validation group. **(C)** Calibration curve for the training group. **(D)** Calibration curve for the testing set.

Discrimination: The AUC reflects the model’s discriminative ability, yielding values of 0.838 (95% CI: 0.777, 0.899) for the training set and 0.723 (95% CI: 0.601, 0.845) for the testing set (**Figures 3A, B**). These findings suggest comparatively strong performance across both datasets, with the training set exhibiting a superior ability to distinguish between outcomes. Importantly, an AUC exceeding 0.7 indicates that the model effectively identifies individuals at both high and low risk of suicide. In the training group, sensitivity is measured at 0.722 and specificity at 0.827, reflecting a commendable balance in the detection of at-risk and non-at-risk individuals. Conversely, in the validation group, sensitivity decreases to 0.686 and specificity to 0.636; nonetheless, these values remain within an acceptable range, suggesting that the model maintains strong applicability across different data sets.

Calibration: The alignment between predicted and observed risks was assessed using calibration curves, demonstrating relatively high predictive accuracy for both training and testing sets (**Figures 3C, D**). The Hosmer-Lemeshow test yielded χ²= 4.436, P = 0.816 for the training set and χ²= 8.393, P = 0.396 for the testing set, indicating a good fit with no significant calibration error.

**Figure 4:**
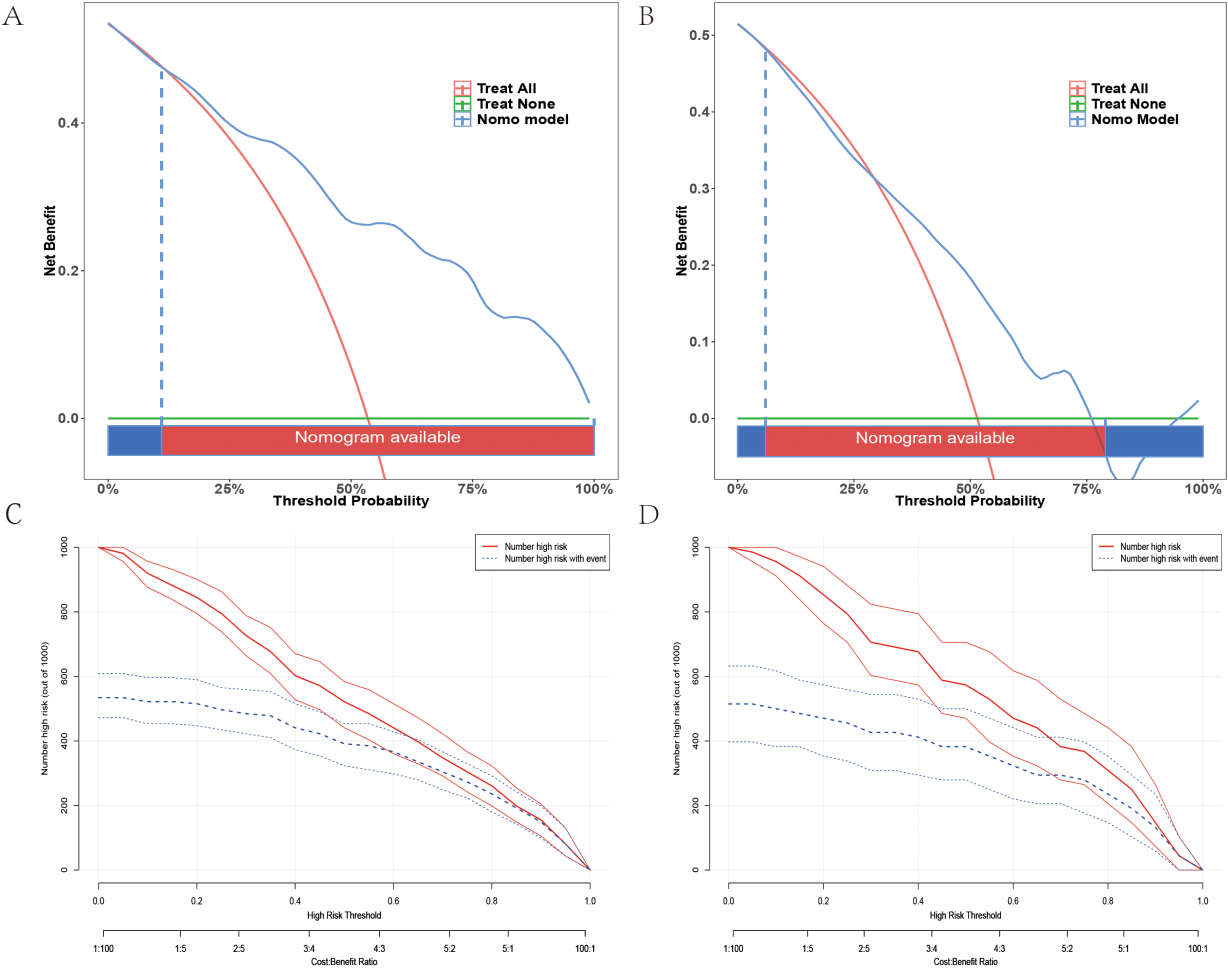
Decision Curve Analysis (DCA) and Clinical Impact Curves. **(A)** DCA curve for the training set. **(B)** DCA curve for the testing set. **(C)** Clinical impact curve for the training set. **(D)** Clinical impact curve for the testing set.

Clinical Applicability: DCA analysis revealed that the model provided higher net benefits across all risk thresholds within the training set (**Figure 4A**). In the testing set, the model exhibited a significantly greater net benefit within the 0%–50% threshold range, thereby supporting its utility in identifying low-to-moderate-risk individuals (**Figure 4B**). Furthermore, the CIC analysis indicated that when the threshold exceeded 0.4, the predicted number of high-risk individuals closely aligned with the actual observed cases. This suggests that the model is particularly valuable for decision-making in the management of high-risk patients (**Figures 4C, D**).

**Figure 5:**
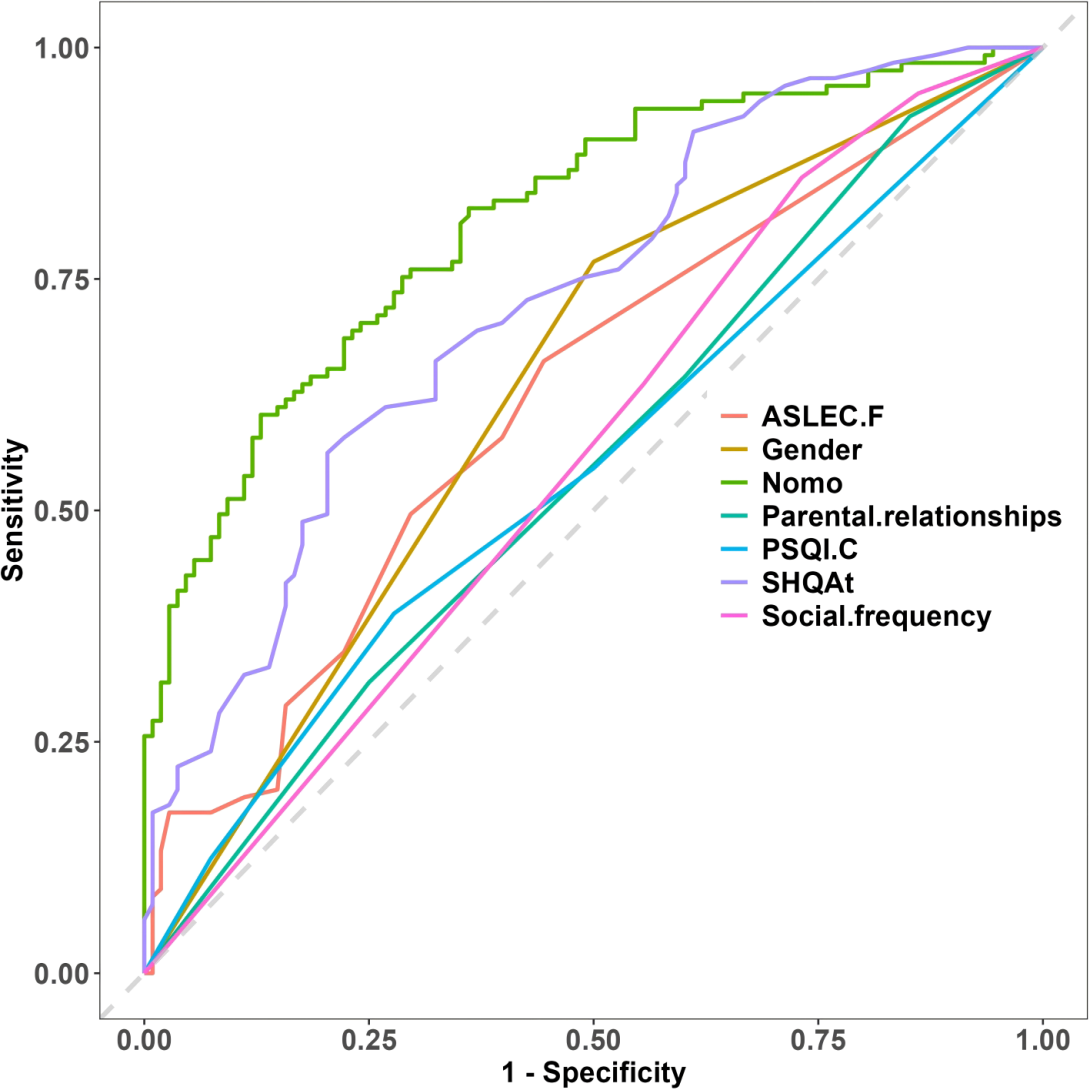
Comparison of nomogram with individual indicators.

Model Comparison and Discriminative Ability: To further evaluate the model’s predictive accuracy, we compared the nomogram-based risk model with a single predictor approach. As illustrated in **Figure 5**, our model outperformed single-indicator models in distinguishing suicide risk, thereby underscoring its enhanced discriminative capability and clinical relevance.

## 4 Discussion

The high incidence of suicide among adolescents with depression highlights the urgent need for early identification and effective intervention strategies. This study employed Lasso regression and multivariate analysis to improve the accuracy of suicide risk prediction by selecting several independent predictors, including gender, social frequency, parental relationships, self-harm behavior, experiences of loss, and insufficient sleep duration. These identified factors were integrated into a predictive model, which was subsequently visualized using a nomogram to facilitate intuitive and practical application. The model demonstrated relatively high discrimination and predictive accuracy, with AUC values of 0.839 in the training set and 0.723 in the testing set. This model enables clinicians to identify high-risk individuals and tailor intervention strategies based on specific scores, thereby promoting targeted prevention efforts. Furthermore, it provides families of patients with insights into relevant risk factors, establishing a scientific foundation for subsequent interventions.

### 4.1 Self-harm behavior as a warning signal for suicide

Self-harm is a well-established predictor of suicide risk in depressed adolescents (66). In this study, self-harm behavior was significantly associated with an increased suicide risk (OR = 1.06, 95% CI: 1.02–1.10, P < 0.001), reinforcing its role as a key warning sign, and indicating its critical association with identifying individuals at heightened risk. This finding is consistent with prior research showing that non-suicidal self-injury (NSSI) is a strong predictor of future suicidal thoughts and attempts (67).

Self-harm is often employed as a maladaptive coping strategy to manage overwhelming distress, particularly in adolescents with limited emotional regulation skills (68). In the short term, it may provide relief; however, longitudinal studies indicate that repetitive self-harm significantly increases the risk of transitioning to suicidal behavior, especially in individuals experiencing social isolation or depression (69, 70).

Our study reinforces this evidence, demonstrating that adolescents who engage in self-harm are at significantly higher risk of suicide. This association is particularly relevant in the Chinese context, where academic stress and cultural expectations may exacerbate emotional distress, leading to higher rates of self-injurious behavior (H. 71, 72). Therefore, addressing self-harm in clinical assessments is crucial for early intervention and suicide prevention.

Given the strong link between self-harm and suicide risk, early intervention is essential. Evidence-based therapies such as Cognitive-Behavioral Therapy (CBT) and Dialectical Behavior Therapy (DBT) effectively reduce self-harm and improve distress tolerance in adolescents (73, 74). Importantly, integrating routine self-harm screening into adolescent depression assessments could enhance early detection and facilitate timely intervention, potentially lowering long-term suicide risk.

### 4.2 Gender differences and suicide risk

Our study reveals that female adolescents with depression are significantly more vulnerable to suicide risk compared to their male counterparts (OR = 2.91, 95% CI: 1.25–7.07, P = 0.015). This finding aligns with existing research indicating that females are more likely to exhibit internalizing symptoms, such as depression and anxiety, while males tend to display externalizing behaviors, including impulsivity and aggression (75). These gender differences highlight distinct mechanisms of suicide risk, emphasizing the need for tailored prevention strategies.

In China, sociocultural factors may further amplify this disparity. Adolescent girls are often socialized to conform to traditional expectations of emotional restraint and compliance, which may reduce their likelihood of expressing distress or seeking psychological help (76). Furthermore, greater emotional sensitivity and rumination tendencies in females have been associated with increased suicidal ideation and behaviors (77, 78).

While male adolescents demonstrate a lower overall risk of suicide attempts, studies suggest that they may be more prone to impulsive decision-making, potentially leading to higher lethality in suicide methods (79). This indicates that males at risk of suicide may require interventions that emphasize impulse control and alternative coping strategies.

These findings underscore the necessity for gender-sensitive interventions. Encouraging emotional expression, addressing gender-specific distress, and expanding counseling services may help reduce suicide risk in female adolescents. For male adolescents, targeted interventions focusing on emotion regulation, distress tolerance, and impulse control may be particularly beneficial (80). Schools and mental health professionals should implement structured emotional support programs, such as peer mentoring and group therapy, to provide a safe space for adolescents to share their emotions.

### 4.3 The protective role of social frequency in mental health

This study demonstrates that frequent social engagement serves as a protective factor against suicide risk among adolescents with depression (OR = 1.91, 95% CI: 1.25–3.04, P = 0.004). Adolescents who engage in social interactions almost daily exhibit the lowest risk, while the risk increases progressively with a decline in the frequency of social engagement. This underscores the importance of consistent social interaction in suicide prevention, as interpersonal connections provide emotional support, help regulate distress, and prevent excessive rumination (81).

Moreover, our study emphasizes that low social frequency remains a significant predictor of suicide risk even after adjusting for other factors. This suggests that social withdrawal itself contributes to heightened vulnerability rather than merely being a symptom of depression. Reduced social interaction limits access to emotional support, which is crucial for regulating stress-related brain activity and mitigating maladaptive coping strategies (82, 83).

In China, academic pressure frequently restricts adolescents’ opportunities for social engagement, particularly in high school (84). Heavy study schedules, parental expectations, and competitive environments contribute to social withdrawal, further reinforcing emotional distress and increasing psychological isolation (85, 86).

As face-to-face social interactions decrease, adolescents may increasingly rely more on internal coping mechanisms, which are often insufficient for managing stress, thereby amplifying their psychological vulnerability. Given our findings, schools and families should actively facilitate structured peer interactions and social support networks. Interventions such as extracurricular activities, peer mentoring programs, and structured group engagement may help reduce social withdrawal and its associated risks (86).

### 4.4 Influence of parental relationship on suicide risk

Tense parental relationships significantly elevate the risk of suicide among adolescents experiencing depression (OR = 2.65, 95% CI: 1.69–4.41, P < 0.001). A supportive family environment provides adolescents with emotional security, while parental conflict can heighten psychological stress and feelings of isolation, exacerbating depression and increasing the risk of suicide (87).

In Chinese culture, family harmony is regarded as a cornerstone of mental health, and discord between parents can profoundly affect the mental well-being of adolescents. Studies suggest that family-based interventions and improved parent-child communication can significantly reduce the suicide risk in at-risk adolescents (88).

Our findings emphasize the importance of integrating family-based approaches into suicide prevention programs. Parent training programs, conflict resolution workshops, and guided family counseling sessions can foster a healthier family dynamic, thereby mitigating stress-related suicidal behavior in adolescents.

### 4.5 Experience of loss and suicide risk

This study found that experiencing loss significantly increases the risk of suicide among adolescents with depression (OR = 1.25, 95% CI: 1.09–1.47, P = 0.002). Loss events, such as the death of a loved one, parental separation, or the loss of close friendships, can trigger intense emotional distress and feelings of helplessness, particularly in adolescents with underdeveloped emotional regulation skills (89).

Our findings support previous research indicating that unresolved grief and emotional suppression can heighten suicide risk (90). In Chinese families, emotional responses to loss are often overlooked, as academic performance is frequently prioritized over emotional well-being (91). This cultural tendency may discourage adolescents from seeking emotional support, exacerbating their distress and increasing the risk of suicide (92).

To mitigate the impact of loss on adolescent suicide risk, prevention programs should incorporate grief-focused interventions, including expressive therapy, peer support groups, and psychological counseling (93, 94). Additionally, schools and families should foster emotional validation and provide structured support to help adolescents navigate significant life changes (95, 96).

### 4.6 Sleep duration and suicide risk

Adolescents who sleep for at least 7 hours per night have a 28% lower risk of suicide (OR = 0.72, 95% CI: 0.56–0.92, P = 0.009), confirming the protective role of sufficient sleep. Adequate sleep is crucial for emotional regulation and psychological resilience, while chronic sleep deprivation exacerbates anxiety, depression, and suicidal ideation (X. 97). Our findings indicate that adolescents who sleep for less than 7 hours per night face a significantly elevated risk of suicide, underscoring the importance of sufficient sleep in suicide prevention.

In China, intense academic pressure often compels adolescents to sacrifice sleep, which in turn heightens psychological stress and emotional instability (H. 98). Research indicates that sleep deprivation disrupts cognitive function and emotional regulation, making individuals more susceptible to suicidal thoughts (E. B. 99).

In light of our findings, it is imperative for schools and families to implement practical measures to foster better sleep habits. These measures may include establishing consistent sleep schedules, encouraging screen-free wind-down periods before bedtime, and enhancing students’ time management skills to effectively balance study and rest. Creating a structured yet flexible routine may help adolescents maintain adequate sleep without compromising their academic responsibilities.

### 4.7 Limitations and future research directions

While this study provides valuable insights into suicide risk prediction among hospitalized adolescents with depression, several limitations warrant consideration. First, as a single-center study, the model’s generalizability remains limited, emphasizing the need for external validation in multicenter cohorts. Second, the reliance on self-report measures introduces potential recall bias and subjective reporting errors, highlighting the necessity of integrating objective biomarkers or longitudinal assessments to enhance prediction accuracy. Third, the model exhibited a lower AUC in the testing set (0.723) compared to the training set (0.839), suggesting potential overfitting, which could be mitigated by utilizing larger datasets and alternative modeling approaches.

Additionally, while this study demonstrates promising predictive performance, we acknowledge that direct comparisons with other existing suicide risk models were not conducted. Future research should include such comparisons to contextualize the model’s effectiveness relative to alternative predictive frameworks, particularly those based on machine learning. Furthermore, the cross-sectional design precludes causal inference, underscoring the need for longitudinal studies to monitor changes in suicide risk over time. Lastly, since the model was developed within a Chinese adolescent population, its applicability across different cultural contexts remains uncertain, highlighting the importance of cross-cultural validation. Addressing these limitations will be essential for enhancing the model’s robustness and clinical utility.

## 5 Conclusion

This study presents a nomogram-based suicide risk prediction model for hospitalized adolescents with depression, demonstrating strong predictive performance and clinical applicability. While the model offers a valuable tool for individualized risk assessment, further validation is necessary to confirm its robustness across diverse populations and clinical settings. Future research should prioritize multicenter external validation and longitudinal assessment to ultimately refine suicide prevention strategies that better support at-risk adolescents.

## Data Availability

The original contributions presented in the study are included in the article/**Supplementary Material**. Further inquiries can be directed to the corresponding author.
