# Supplementary material for: Developing a suicide risk prediction model for hospitalized adolescents with depression in China
Source: Front Psychiatry. 2025 May 2;16:1532828. doi: 10.3389/fpsyt.2025.1532828 (PMC12081462; doi:10.3389/fpsyt.2025.1532828)
Supplement: Supplementary file 1 [file DataSheet1.pdf]

Supplementary Table S1. Skewness and Kurtosis values with Z-scores for continuous variables (n = 229)

Variable	Skewness	SE (Skewness)	Z-score (Skewness)	Kurtosis	SE (Kurtosis)	Z-score (Kurtosis)
Height	0.61	0.16	3.77	0.26	0.32	0.80
BMI	0.82	0.16	5.09	0.54	0.32	1.69
AGEn	0.10	0.16	0.59	-0.95	0.32	-2.96
SHQA.t	0.36	0.16	2.22	-0.51	0.32	-1.59
SCSQ.AR	0.69	0.16	4.27	0.84	0.32	2.63
SCSQ.NC	-0.04	0.16	-0.22	-0.36	0.32	-1.12
sds.Total	0.69	0.16	4.26	0.32	0.32	1.00
ASLEC.IR	-0.11	0.16	-0.67	-0.70	0.32	-2.20
ASLEC.SP	0.15	0.16	0.95	-0.57	0.32	-1.77
ASLEC.P	0.81	0.16	5.03	0.08	0.32	0.24
ASLEC.F	1.50	0.16	9.34	1.57	0.32	4.89
ASLEC.HA	1.03	0.16	6.37	0.79	0.32	2.46
ASLEC.O	0.29	0.16	1.81	-0.03	0.32	-0.08
Link.DDS	-0.36	0.16	-2.21	1.38	0.32	4.32
Link.SPPS.P	0.18	0.16	1.13	-0.05	0.32	-0.17
Link.SPPS.F	-0.03	0.16	-0.18	0.38	0.32	1.19
Link.SPPS.E	0.18	0.16	1.13	0.00	0.32	-0.01
Link.SPPS.C	0.51	0.16	3.16	0.47	0.32	1.48
Link.SPPS.SE	-0.13	0.16	-0.82	-0.03	0.32	-0.09
Link.SAES.M	0.69	0.16	4.29	1.66	0.32	5.18
Link.SAES.DS	-0.19	0.16	-1.16	-0.17	0.32	-0.54
GSES	0.85	0.16	5.27	1.39	0.32	4.34
DMSC.SA.ISI	0.19	0.16	1.17	-0.82	0.32	-2.56
DMSC.SA.ISD	-0.59	0.16	-3.66	-0.01	0.32	-0.04
DMSC.SA.ISDG	0.30	0.16	1.85	-0.22	0.32	-0.70
DMSC.SA.CSPS	-0.23	0.16	-1.45	0.31	0.32	0.97
DMSC.SA.CSFT	0.59	0.16	3.68	-0.31	0.32	-0.97
DAS.V	-0.17	0.16	-1.07	-0.01	0.32	-0.04
DAS.AR	-0.44	0.16	-2.75	0.47	0.32	1.46
DAS.P	-0.33	0.16	-2.07	-0.31	0.32	-0.98
DAS.M	-0.16	0.16	-0.97	0.03	0.32	0.10
DAS.AT	-0.11	0.16	-0.70	-0.19	0.32	-0.59
DAS.D	-0.24	0.16	-1.52	0.00	0.32	-0.01
DAS.AA	-0.38	0.16	-2.35	-0.75	0.32	-2.34
DAS.CP	-0.11	0.16	-0.66	-0.38	0.32	-1.19
CD.RISC.T	0.61	0.16	3.80	0.68	0.32	2.13
CD.RISC.C	0.56	0.16	3.49	0.83	0.32	2.60
CD.RISC.O	0.46	0.16	2.88	0.29	0.32	0.92
FACESII.CV.I	0.15	0.16	0.94	-0.34	0.32	-1.06
FACESII.CV.A	0.26	0.16	1.64	-0.22	0.32	-0.67
MFI.20.GF	0.04	0.16	0.27	0.56	0.32	1.76

MFI.20.PF	0.47	0.16	2.93	0.17	0.32	0.52
MFI.20.MF	0.40	0.16	2.50	-0.08	0.32	-0.23
MFI.20.RA	0.32	0.16	2.00	0.07	0.32	0.22
MFI.20.DM	-0.11	0.16	-0.66	-0.18	0.32	-0.55
PBI.I.F	0.19	0.16	1.19	-0.66	0.32	-2.08
PBI.I.M	0.17	0.16	1.06	-0.33	0.32	-1.02
PBI.II.F	0.85	0.16	5.30	0.22	0.32	0.69
PBI.II.M	0.43	0.16	2.66	-0.37	0.32	-1.16
PBI.III.F	0.45	0.16	2.78	-0.24	0.32	-0.74
PBI.III.M	0.55	0.16	3.39	-0.21	0.32	-0.66
PBI.IV.F	0.49	0.16	3.06	-0.63	0.32	-1.98
PBI.IV.M	0.83	0.16	5.15	0.50	0.32	1.57
PBI.V.F	0.64	0.16	3.98	0.32	0.32	1.01
PBI.V.M	0.29	0.16	1.81	-0.89	0.32	-2.78
PBI.VI.F	0.29	0.16	1.81	-0.36	0.32	-1.11
FAD.PS	-0.10	0.16	-0.60	1.00	0.32	3.11
FAD.C	-0.21	0.16	-1.31	0.46	0.32	1.43
FAD.R	-0.50	0.16	-3.12	1.13	0.32	3.53
FAD.ER	-0.26	0.16	-1.63	0.35	0.32	1.10
FAD.EI	-0.18	0.16	-1.14	0.54	0.32	1.67
FAD.BC	-0.64	0.16	-3.98	1.56	0.32	4.88
FAD.TF	-0.28	0.16	-1.71	1.18	0.32	3.70
PSQI.A	0.30	0.16	1.86	-0.53	0.32	-1.65
PSQI.B	-0.47	0.16	-2.92	-0.08	0.32	-0.25
PSQI.C	0.60	0.16	3.70	-1.04	0.32	-3.25
PSQI.D	1.33	0.16	8.25	0.34	0.32	1.05
PSQI.E	0.20	0.16	1.22	-0.28	0.32	-0.88
PSQI.F	0.75	0.16	4.65	-0.81	0.32	-2.54
PSQI.G	0.33	0.16	2.02	-1.24	0.32	-3.86
FES.CV.INT	0.05	0.16	0.31	-1.01	0.32	-3.16
FES.CV.EE	0.30	0.16	1.84	-0.82	0.32	-2.56
FES.CV.CON	-0.31	0.16	-1.91	-0.86	0.32	-2.68
FES.CV.IND	0.08	0.16	0.47	-0.30	0.32	-0.94
FES.CV.S	-0.28	0.16	-1.75	-0.10	0.32	-0.32
FES.CV.CUL	0.46	0.16	2.88	-0.45	0.32	-1.41
FES.CV.E	0.57	0.16	3.52	-0.51	0.32	-1.60
FES.CV.MRO	-0.02	0.16	-0.15	-0.37	0.32	-1.17
FES.CV.O	-0.08	0.16	-0.48	-0.69	0.32	-2.15
FES.CV.Control	0.33	0.16	2.02	-0.59	0.32	-1.83
SSRS.OSP	0.57	0.16	3.55	0.69	0.32	2.15
SSRS.SSP	0.26	0.16	1.64	-0.40	0.32	-1.26
SSRS.US	0.79	0.16	4.89	0.72	0.32	2.23
PSCS.T	0.12	0.16	0.72	-0.14	0.32	-0.44
PSCS.P	0.07	0.16	0.44	1.44	0.32	4.51
PSCS.A	0.53	0.16	3.29	-0.04	0.32	-0.13

Note: Variables with skewness or kurtosis Z-scores beyond ±1.96 were considered non-normally distributed. Detailed assessment guided the choice of parametric or non-parametric tests in the analysis.
